# Cerebral ischemia induces microvascular pro-inflammatory cytokine expression via the MEK/ERK pathway

**DOI:** 10.1186/1742-2094-8-18

**Published:** 2011-02-21

**Authors:** Aida Maddahi, Lars Edvinsson

**Affiliations:** 1 Division of Vascular Research, BMC, Lund University, Lund, Sweden; 2 Department of Internal Medicine, Institute of Clinical Sciences, Lund University, Lund, Sweden

## 

We wish to clarify in results page 4, line 22 of our study [1]; that the information on infarct volume and neurology score after MCAO was taken from our previous study [2].
